# *Lycium barbarum* Polysaccharide Prevents Focal Cerebral Ischemic Injury by Inhibiting Neuronal Apoptosis in Mice

**DOI:** 10.1371/journal.pone.0090780

**Published:** 2014-03-03

**Authors:** Tengfei Wang, Yuxiang Li, Yongsheng Wang, Ru Zhou, Lin Ma, Yinju Hao, Shaoju Jin, Juan Du, Chengjun Zhao, Tao Sun, Jianqiang Yu

**Affiliations:** 1 Department of Pharmacology, Ningxia Medical University, Yinchuan, People's Republic of China; 2 College of Nursing, Ningxia Medical University, Yinchuan, People's Republic of China; 3 Key Lab of Craniocerebral Diseases of Ningxia Hui Autonomous Region, Ningxia Medical University, Yinchuan, People's Republic of China; 4 Key Laboratory of Fertility Preservation and Maintenance, Ningxia Medical University, Yinchuan, People's Republic of China; 5 Ningxia Hui Medicine Modern Engineering Research Center, Ningxia Medical University, Yinchuan, People's Republic of China; National University of Singapore, Singapore

## Abstract

**Aims of the Study:**

To investigate the neuroprotective effect of *Lycium barbarum* polysaccharide (LBP) on focal cerebral ischemic injury in mice and to explore its possible mechanism.

**Materials and Methods:**

Male ICR mice were used to make the model of middle cerebral artery occlusion (MCAO) after intragastric administration with LBP (10, 20 and 40 mg/kg) and Nimodipine (0.4 mg/kg) for seven successive days. After 24 h of reperfusion, neurological scores were estimated and infarct volumes were measured by 2, 3, 5-triphenyltetrazolium chloride (TTC) staining. Morphological changes in ischemic brains were performed for hematoxylin-eosin (HE) staining. The number of apoptotic neurons was detected by TUNEL staining. The Bax, Bcl-2 protein expression and CytC, Caspase-3, -9 and cleaved PARP-1 activation were investigated by immunofluorescence and western-blot analysis.

**Results:**

LBP (10, 20 and 40 mg/kg) treatment groups significantly reduced infract volume and neurological deficit scores. LBP also relieved neuronal morphological damage and attenuated the neuronal apoptosis. LBP at the dose of 40 mg/kg significantly suppressed overexpression of Bax, CytC, Caspase-3, -9 and cleaved PARP-1, and inhibited the reduction of Bcl-2 expression.

**Conclusions:**

Based on these findings we propose that LBP protects against focal cerebral ischemic injury by attenuating the mitochondrial apoptosis pathway.

## Introduction

Ischemic stroke is a major cause of human death and disability worldwide [1]. In the process of cerebral ischemia, a cascade of pathological mechanisms including excessive release of excitatory amino acids, energy failure, increased oxidative stress and apoptosis will be activated, eventually resulting in acute cerebral ischemic injury [2], [3]. Apoptosis is one of the primary factors in cell death after cerebral ischemia reperfusion, it is an initiative suicide process after the cells receive related signals [4]. There are two major pathways of apoptosis after cerebral ischemia: the intrinsic pathway and the extrinsic pathway, the intrinsic pathway, also called mitochondrial apoptosis pathway, is originated from mitochondrial release of cytochromec (CytC) and associated stimulation of caspase-3 [5].

In the intrinsic pathway, release of CytC leads to the formation of the apoptosome and then promotes the activation of procaspase-9 [6]. The clustering of procaspase-9 leads to caspase-9 activation. Caspase-9, which is considered as an initiator of the mitochondria-dependent caspase cascade, then activates caspase-3 [7]. Caspase-3 can cleave many substrate proteins, such as poly (ADP-ribose) polymerase (PARP) [8], [9]. Overactivation of PARP after cleavage by caspase-3 leads to DNA injury and subsequently to apoptotic cell death [5]. On the other hand, apoptosis is also regulated by a series of protein systems, one of these systems is the Bcl-2 family [10], [11]. Bax, as a major proapoptotic protein in the Bcl-2 family, plays a key role in promoting apoptosis [12], [13]. Bcl-2 protein is an important inhibitor of apoptosis which prevents the release of CytC and Caspase activation [14], [15].

*Lycium barbarum* polysaccharide (LBP), a major active ingredient of *Lycium barbarum*, has been reported to have several pharmacological activities such as anti-oxidative [16], anti-proliferate [17] and hypoglycemic effect [18]. Our previous study demonstrated that LBP has protective effects on hippocampus neurons model of ischemic cerebral injury in vitro [19]. In the present study, we examined the protective effects of LBP in a model of middle cerebral artery occlusion (MCAO) in mice. Furthermore, we investigated the relations between the effects of LBP and the mitochondrial apoptosis pathway in mice brains after stroke. Nimodipine, as a classic clinic drug in stroke, has shown obviously beneficial effects on active treatment in previous studies [20], [21]. So it was also used as a positive control in this study.

## Results and Discussion

### LBP provided neuroprotection after cerebral ischemia in MCAO mice

**Figure 1:**
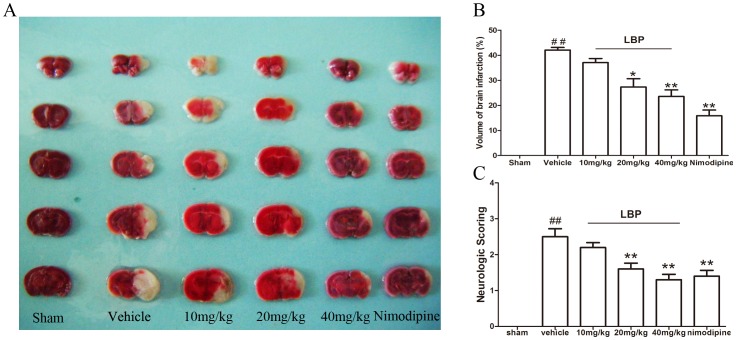
Protective effect of LBP against cerebral ischemic injury in MCAO mice brains. **A** TTC staining of representative coronal sections at 24**B** Quantitative analysis of infarct volumes at 24 h after reperfusion. **C** Quantification of neurologic scores at 24 h after reperfusion. Data are expressed as mean±SEM (n = 6). ^##^P<0.01, vs. sham-operated group, * P<0.05, **P<0.01 vs. vehicle group.

In the representative photographs of TTC staining (Figure 1A), normal brain tissues appeared uniform red while the infarction region showed white. There was no infarct found in the sham mice brain and an obvious infarcted area appeared in the vehicle group. The infarction regions were significantly decreased after MCAO in LBP (20 and 40 mg/kg, i.g.) groups (p<0.05) and Nimodipine group (P<0.01) compared with vehicle group (Figure 1B).

The neurological deficits could be observed in mice after cerebral ischemic injury. As shown in Figure 1C, the neurological deficits were significantly increased in the vehicle-ischemic group compared to sham-operated group (P<0.01). However, after treatment with LBP and Nimodipine, the neurological deficit scores were reduced respectively, and the effects of LBP (20, 40 mg/kg, i.g.) groups and Nimodipine (0.4 mg/kg, i.g.) groups were significant (P<0.01).

**Figure 2:**
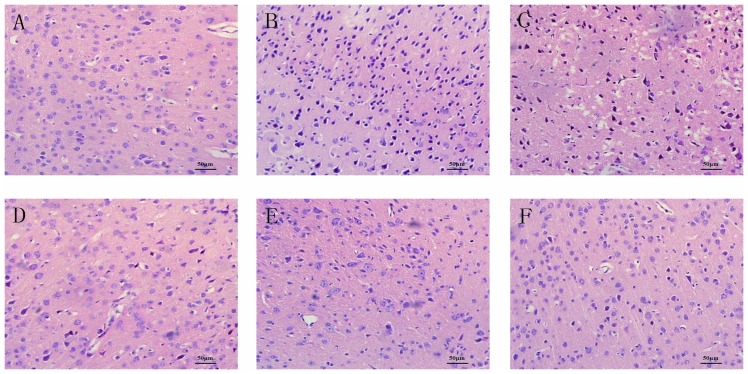
Effect of LBP treatment on morphological changes in ischemic penumbra of the cortex at 24-eosin staining (400×). A Sham-operated group. B Vehicle group. C LBP 10 mg/kg group. D LBP 20mg/kg group. E LBP 40mg/kg group. F Nimodipine 0.4mg/kg group.

The HE staining could visually show the histological changes in neurons of mice brains in different groups (Figure 2). In the sham-operated group, morphology of neurons in ischemic penumbra of the cortex was round or oval in shape and the nucleus were clear. After MCAO, a large number of apoptotic neurons with karyopyknosis, cell gaps and debris could be observed in vehicle group. LBP (10, 20, 40 mg/kg, i.g.) groups and Nimodipine group alleviated the symptoms of apoptosis in different degrees.

All of these findings indicated that LBP reduced brain injury induced by cerebral ischemia. In addition, these results revealed that the neuroprotective effect in the 40 mg/kg LBP group is more obvious than that in 10 and 20 mg/kg LBP groups.

### Effects of LBP on TUNEL-positive cells after cerebral ischemic injury

**Figure 3:**
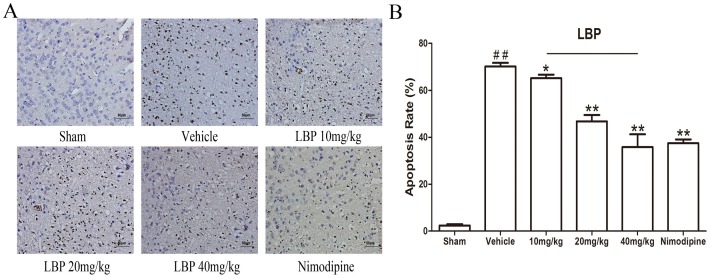
LBP reduces the number of Tunel positive neurons after focal cerebral ischemic injury. **A** TUNEL staining of representative sections in mice ischemic penumbra of the cortex at 24**B** Quantitative analysis of apoptosis cells in cortex in different groups at 24 h after reperfusion. Data are expressed as mean±SEM (n = 6). ^##^P<0.01 vs. sham-operated group; *P<0.05, **P<0.01 vs. vehicle group.

We next examined whether LBP helped to prevent cerebral ischemia-induced DNA fragmentation. In the TUNEL staining, the TUNEL-positive cells were darkly stained and showed the morphologic signs of apoptosis (Figure 3A). In the vehicle group, the apoptosis rate in ischemic penumbra of the left cortex was significantly increased. Treatment with LBP significantly reduced the number of TUNEL- positive cells compared with vehicle group after 24h of reperfusion, and the apoptosis rate was reduced obviously in LBP (10, 20, 40mg/kg) groups and Nimodipine group respectively (Figure 3B, P<0.05).

### LBP influenced the expression of apoptosis related factors

**Figure 4:**
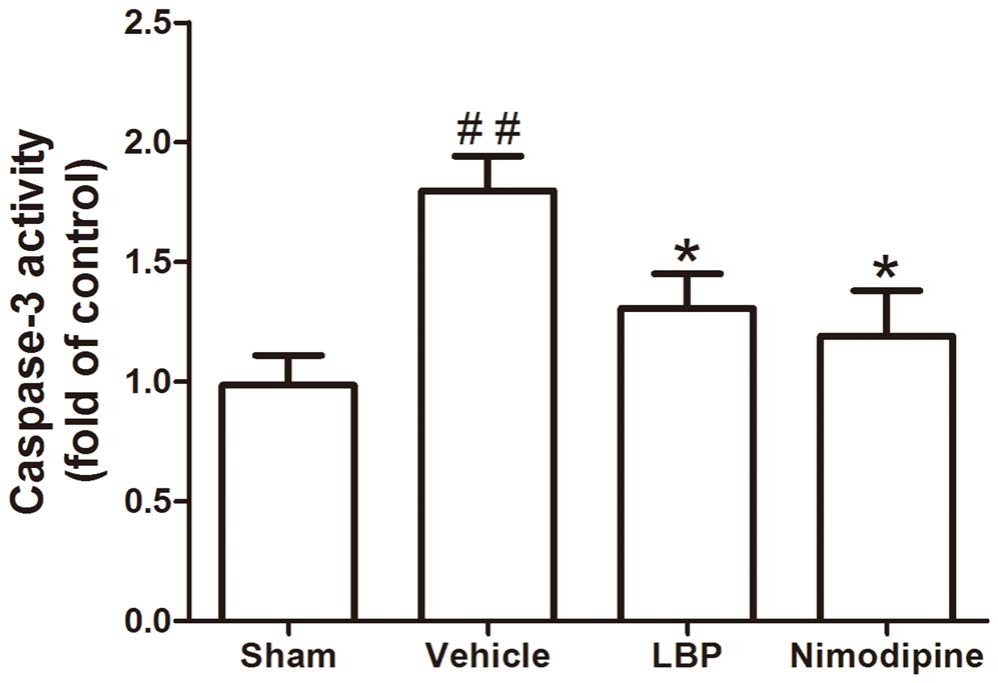
Effects of LBP (40 mg/kg) on the caspase-3 activities in left hemisphere after 2 h of MCAO and 24 h of reperfusion. Data are expressed as mean±SEM (n = 6). ^##^P<0.01, vs. sham-operated group, *P<0.05 vs. vehicle group.

The caspase-3 activity in ischemic hemisphere of vehicle group increased significantly compared with sham-operated group (P<0.01). The activities of caspase-3 were markedly reduced in LBP 40 mg/kg treatment group and Nimodipine group (Figure 4; both P<0.05).

**Figure 5:**
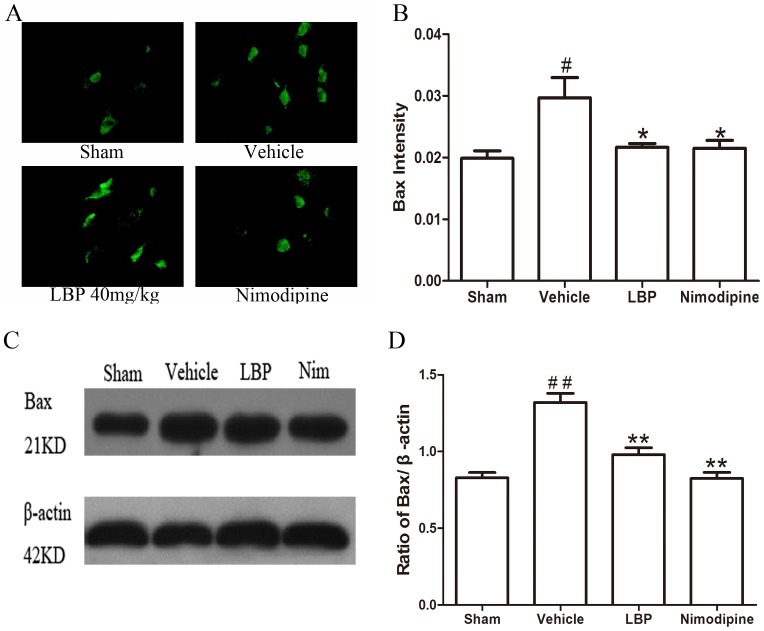
Effects of LBP on the expression of Bax protein. **A** Representative photomicrographs of Bax immunofluorescence staining (400×). **B** Quantification of Bax protein fluorescence intensity in different groups. **C** Representative Western blot band of Bax protein expression in the ischemic cortex at 24 h after reperfusion. **D** Effect of LBP (40 mg/kg) on the Bax expression in MCAO mice cortex at 24 h after reperfusion. Data are expressed as mean±SEM (n = 6). ^#^P<0.05, ^##^P<0.01 vs. sham-operated group; *P<0.05, **P<0.01 vs. vehicle group.

**Figure 6:**
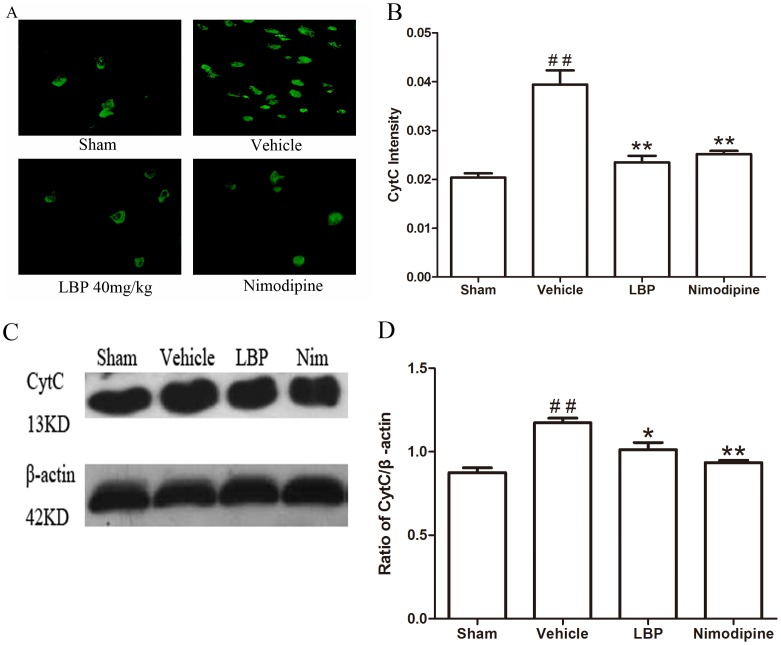
Effects of LBP on the expression of CytC. **A** Representative photomicrographs of CytC immunofluorescence staining (400×). **B** Quantification of CytC fluorescence intensity in different groups. **C** Representative Western blot band of CytC activation in the ischemic cortex at 24 h after reperfusion. **D** Effect of LBP (40 mg/kg) on the CytC activation in MCAO mice cortex at 24 h after reperfusion. Data are expressed as mean±SEM (n = 6). ^##^P<0.01 vs. sham-operated group; *P<0.05, **P<0.01 vs. vehicle group.

**Figure 7:**
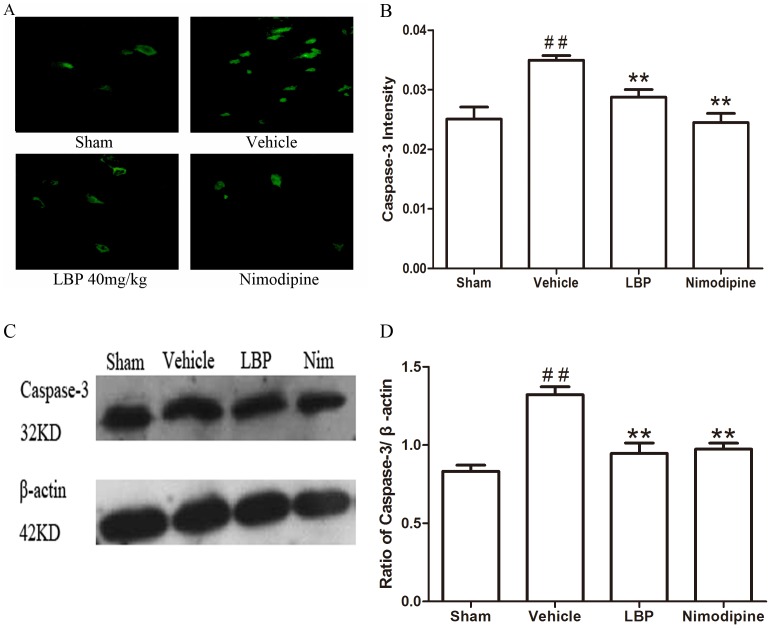
Effects of LBP on the expression of Caspase-3. **A** Representative photomicrographs of Caspase-3 immunofluorescence staining (400×). **B** Quantification of Caspase-3 fluorescence intensity in different groups. **C** Representative Western blot band of Caspase-3 activation in the ischemic cortex at 24 h after reperfusion. **D** Effect of LBP (40 mg/kg) on the Caspase-3 activation in MCAO mice cortex at 24 h after reperfusion. Data are expressed as mean±SEM (n = 6). ^##^P<0.01 vs. sham-operated group; **P<0.01 vs. vehicle group.

**Figure 8:**
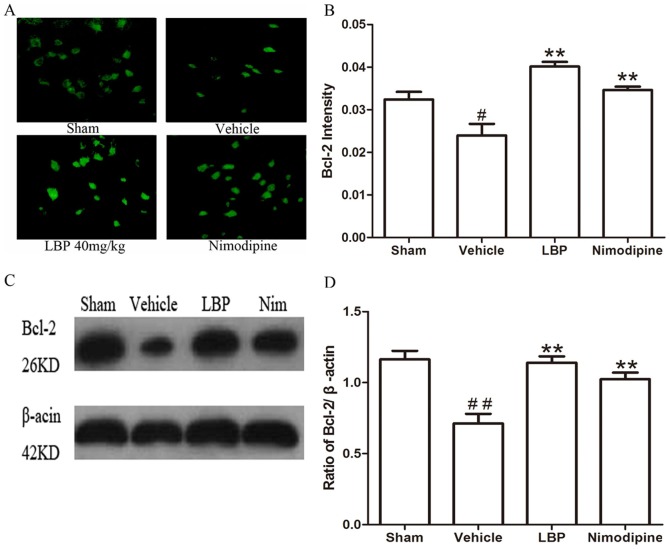
Effects of LBP on the expression of Bcl-2 protein. **A** Representative photomicrographs of Bcl-2 immunofluorescence staining (400×). **B** Quantification of Bcl-2 protein fluorescence intensity in different groups. **C** Representative Western blot band of Bcl-2 protein expression in the ischemic cortex at 24 h after reperfusion. **D** Effect of LBP (40 mg/kg) on the Bcl-2 expression in MCAO mice cortex at 24 h after reperfusion. Data are expressed as mean±SEM (n = 6). ^#^P<0.05, ^##^P<0.01 vs. sham-operated group; **P<0.01 vs. vehicle group.

In the Immunofluorescence assay, the fluorescence intensity showed the activations of apoptosis related factors. Results of immunohistochemistry showed that the fluorescence intensity of Bax protein and CytC, Caspase-3 were significantly higher in the vehicle group than sham-operated group (P<0.05), and these increase were significantly attenuated by LBP 40 mg/kg and Nimodipine groups (Figure 5B, 6B and 7B, P<0.05). The expression of Bcl-2 protein was decreased significantly in vehicle group (p<0.05) compared with sham-operated group. Treatment with 40 mg/kg LBP or 0.4 mg/kg Nimodipine could significantly increase the level of Bcl-2 protein (Figure 8B, p<0.01).

**Figure 9:**
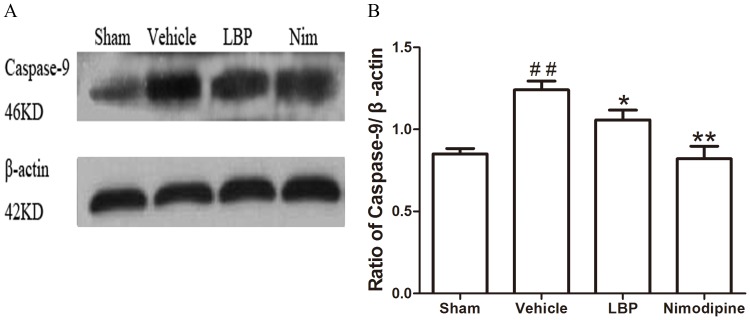
Effects of LBP on the expression of Caspase-9. **A** Representative Western blot band of Caspase-9 activation in the ischemic cortex at 24 h after reperfusion. **B** Effect of LBP (40 mg/kg) on the Caspase-9 activation in MCAO mice cortex at 24 h after reperfusion. Data are expressed as mean±SEM (n = 6). ^##^P<0.01 vs. sham-operated group; *P<0.05, **P<0.01 vs. vehicle group.

**Figure 10:**
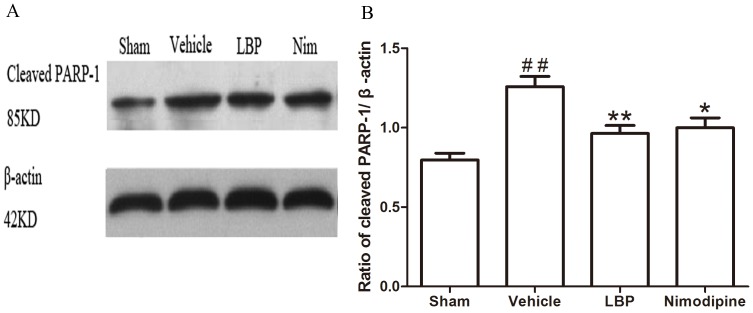
Effects of LBP on the expression of Cleaved PARP-1. **A** Representative Western blot band of Cleaved PARP-1 activation in the ischemic cortex at 24 h after reperfusion. **B** Effect of LBP (40 mg/kg) on the Cleaved PARP-1 activation in MCAO mice cortex at 24 h after reperfusion. Data are expressed as mean±SEM (n = 6). ^##^P<0.01 vs. sham-operated group; *P<0.05, **P<0.01 vs. vehicle group.

In addition, we assessed the Bax, Bcl-2 protein expression and CytC, Caspase-3, -9 and cleaved PARP-1 activation in ischemic brain tissues by Western blot analysis after 24 h of reperfusion. The protein level of Bax was increased while the expression of Bcl-2 protein was decreased significantly in vehicle group compared with sham-operated group. In the LBP 40 mg/kg group and Nimodipine group, expression of Bax was decreased significantly (Figure 5D, p<0.01). LBP 40 mg/kg group and Nimodipine group significantly inhibited the reduction of Bcl-2 expression (Figure 8D, p<0.01). As proapoptotic markers in mitochondrial apoptosis pathway, the activations of CytC, Caspase-3, -9 and cleaved PARP-1 were markedly increased in vehicle group compared with sham-operated group. After treatment with 40 mg/kg LBP or 0.4 mg/kg Nimodipine, the activations of these four apoptotic factors were obviously reduced compared with vehicle group (Figure 6D, 7D, 9B and 10B, p<0.05).

### Mitochondrial apoptosis pathway after cerebral ischemic injury

Ischemic brain disease has become one of the most devastating diseases which cause high rates of disability and mortality in aged persons [22]. Several harmful pathological changes have taken place in the process of cerebral ischemia, which cause a difficult determination of the more appropriate drug target. Then it is a great challenge to find a novel pharmacological drug with protective effect in cerebral ischemic injury. Traditional Chinese Medicines are proved to have many bioactivities which provide neuroprotective effects in ischemic brain disease [23]-[25]. In the present study, we used the method of MCAO-induced cerebral ischemic injury in mice to mimic human cerebral ischemic disease, we further investigated whether LBP had neuroprotective effects in MCAO mice and its pharmacological mechanism related with mitochondrial apoptosis pathway after cerebral ischemic injury.

Previous studies have demonstrated neuronal cell apoptosis plays an important role in the evolution of ischemic injury in the brain tissue [26]. Mitochondrial apoptosis pathway is a major apoptosis pathway, a large number of apoptosis-related proteins are found in mitochondria, including cytochromec (CytC), apoptosisinducingfactor (AIF), procaspase -3, -8 and -9 [27]. Cerebral ischemia can promote a series of pathological changes in neuronal cells such as mitochondrial membrane depolarization and permeability transition pore opening [28]. CytC binds and activates apoptotic protease-activating factor-1 (Apaf-1) as well as procaspase-9, they form an apoptosome together with ATP. Apoptosome then activates Caspase-9 and Caspase-9 leads to Caspase-3 activation, ultimately cell death occurs [29]. Caspase-3 has been identified as a key mediator of apoptosis and cleaves the substrate protein - poly (ADP-ribose) polymerase (PARP). PARP-1 is a multifunctional nuclear enzyme whose activity is rapidly stimulated by DNA breaks [30], overactivation of cleaved PARP-1 can cause DNA fragmentation and cell dysfunctions, and then promote cell death. On the other hand, proapoptotic and antiapoptotic Bcl-2 family proteins are believed to play important roles in mitochondrial apoptosis pathway [31], [32]. Bax is a proapoptotic and Bcl-2 is an antiapoptotic protein in the Bcl-2 family. An appropriate ratio of Bax and Bcl-2 can keep homeostatic state in cells and ensure cell survival.

### Conclusion

In this study, treatment of MCAO mice with LBP could markedly reduce neurological deficit scores and infract volume in ischemic brains. Morphological changes of apoptotic cells induced by MCAO were inhibited in LBP treatment groups respectively. LBP also showed a significant effect to decrease the number of apoptotic neurons. To investigate its possible anti-apoptotic mechanism, we have measured some apoptosis-related proteins especially mitochondrial apoptosis pathway-related proteins. Activity of caspase-3 in ischemic brain of MCAO mice was markedly attenuated after treatment with 40 mg/kg LBP. The results of immunofluorescence and western-blot analysis demonstrated that LBP with the dose of 40 mg/kg could significantly suppress the overexpression of Bax, CytC, Caspase-3, -9 and cleaved PARP-1 and promote Bcl-2 expression in ischemic mice brain cortex.

These results in the present study indicated that LBP had neuroprotective effect in focal cerebral ischemic injury in mice. And the protection of cerebral ischemic mice against MCAO-induced apoptosis by LBP is associated with mitochondria apoptosis pathway.

In this study, the mice focal cerebral ischemic injury was produced by 2 hours of MCAO and reperfusion for 24 hours. The optimal duration of treatment and optimal therapeutic dose of LBP against cerebral ischemic injury were not fully elucidated. Besides mitochondria apoptosis pathway, there is the other major apoptosis pathway, the extrinsic pathway, which originates from the activation of cell surface death receptors, so it needs further research in the future study to investigate the best treatment options of LBP in cerebral ischemic injury.

## Materials and Methods

### Animals and Drug Preparation

Male ICR mice (n = 108) weighed from 20.0 to 25.0 g were afforded by animal centre of Ningxia Medical University. The mice were housed under a 12/12 h dark/light cycle and provided food and water ad libitum. *Lycium barbarum* polysaccharide (LBP) was supplied by Ningxia Agricultural and forest College, and dissolved with physiological saline. The experiments were performed as approved by the institutional animal care and use committee of Ningxia Medical University. All surgery was performed under chloral hydrate anesthesia, and all efforts were made to minimize suffering. All of the mice were randomly divided into the following six groups (n = 18, for each group): sham-operated groups (sham); vehicle-treated ischemic model group (vehicle); 10 mg/kg LBP-treated ischemic group (10 mg/kg); 20 mg/kg LBP-treated ischemic group (20 mg/kg); 40 mg/kg LBP-treated ischemic group (40 mg/kg) and 0.4 mg/kg Nimodipine-treated ischemic group (Nimodipine). LBP and Nimodipine were given by intragastric gavage for seven consecutive days before cerebral ischemia. Sham and vehicle groups were treated with physiological saline under the same conditions.

### Middle Cerebral Artery Occlusion (MCAO) Model

Focal cerebral ischemia was produced by the method as described previously [33] except mice in sham group. After anesthetized with 3.5% chloral hydrate, the mice were used to make a neck incision. Then the left external carotid artery (ECA) and internal carotid artery (ICA) were exposed and separated carefully. A length of monofilament nylon suture (15 mm) was advanced from the ECA into the lumen of the ICA to occlude the origin of the left middle cerebral artery (MCA). After 2 h of ischemia, the filament was withdrawn. Sham-operated group mice were subjected to the same surgical procedure but the MCA was not occluded.

### Evaluation of Neurological Deficits

The neurological deficit scores in each group (n = 6) were performed after 2 h of ischemia and 24 h of reperfusion. Deficits scores were carried out according to a five-point scale adapted from a previous publication [34]. A score of 0 was given if the mouse was demonstrated normal spontaneous movements (no neurological deficit); 1 was given if the mouse was unable to extend right paw fully; 2 was given if the mouse was circling to right; 3 was given if the mouse was falling to right and 4 was given if the mouse was unable to walk spontaneously. All the neurological evaluations were performed by a researcher who was unaware of the different groups.

### Determination of Infarct Volume

The mice (n = 6, for each group) were decapitated to remove the brain after the evaluation of neurological deficit. The ischemic brains were cut into 1 mm sections and the brain slices were stained in 2% solution of 2,3,5,-triphenyl tetrazolium chloride (TTC) (Sigma,St. Louis, MO, U.S.A.) for 30 min at 37°C followed by overnight immersion in 4% formaldehyde solution. The infarct volumes were calculated with microscope image-analysis software (Image-Pro plus, USA). Infarct area of each brain section was added to derive the total infarct areas and the infarct volume was obtained after the total infarct areas being multiplied by the thickness of the sections. The degree of cerebral infarction was presented as the percentage of infarct volume to total brain volume.

### TUNEL staining

DNA fragmentation was performed by In Situ Cell Death Detection Kit (Roche, Germany) and was detected under optical microscope. On brief, mice (n = 6, for each group) were deeply anesthetized by 3.5% choral hydrate after 24 h of reperfusion, then perfused with physiological saline and 4% paraformaldehyde from the heart to the systemic vascular. The brains were collected and embedded in paraffin, coronal sections (5µm thick) were taken from brain and stained according to the methods provided by the manufacturer. Apoptotic cells were stained brown due to the binding of dUTP enzyme to their fragmented DNA. Apoptotic neurons in the ischemic cortex were counted for five fields per section under high-power magnification (400×) by a blinded manner.

### Immunofluorescence analysis and hematoxylin-eosin (HE) staining

Immunofluorescence and hematoxylin-eosin (HE) staining for ischemic brain tissue were performed on paraffin sections which were prepared using the same method as in TUNEL staining. After incubation in 0.5% H_2_O_2_ followed by normal goat serum to avoid nonspecific immunoreactions, the sections (n = 6, for each group) were incubated with primary antibodies of Bax, Bcl-2, CytC and Caspase-3, (Bax, 1:50; Bcl-2, 1:50; Caspase-3, 1:50; Proteintech Group, U.S.A. and CytC, 1:50; Abcam, U.K.) at 4°C overnight. The next day, sections were washed with PBS for 15 min and incubated for 1 h at 37°C with FITC-labeled Goat Anti-Rabbit IgG (1:20; Proteintech Group, U.S.A.). Mean density of Bax, Bcl-2, CytC and Caspase-3 in mice brains per section (400×) was measured with microscope image-analysis software (Image-Pro plus, U.S.A.) by a single investigator who was blind to sample identity.

### Caspase-3 activity assay

Activity of caspase-3 was measured by cleaving selective substrates Ac-DEVD-*p*NA with a caspase-3 activity kit (Beyotime Institute of Biotechnology, China). The brains (n = 6, for each group) were collected, weighed and homogenized in lysis buffer. The homogenate was centrifuged at 20, 000 g and 4°C for 15 min. Substrate cleavage was measured with a microplate reader (Bio-Rad, U.S.A) at 405 nm, and then was corrected as protein content in the lysate. The Caspase-3 activity was expressed as values of enzyme activity compared with control.

### Western blot Analysis

After 24 h of reperfusion, the mice (n = 6, for each group) were decapitated and ischemic brains were collected. Protein lysates of ischemic hemispheres in each group were subjected to 8% or 12% sodium dodecyl sulfate-polyacrylamide gel electrophoresis (SDS-PAGE) and transferred onto a polyvinylidene difluoride (PVDF) membrane. The membrane was treated with blocking solution (5% skim milk in TBST) and incubated overnight at 4°C with the primary rabbit monoclonal antibodies respectively (Bax, 1:1000; Bcl-2, 1:500; Caspase-3, 1:1000; Caspase-9, 1:500; Proteintech Group, U.S.A.; CytC, 1:1000; Abcam, U.K. and PARP-1, 1:500; Cell Signal, U.S.A.). Secondary incubations were performed with horseradish peroxidase-conjugated goat anti-rabbit antibody (1:3000; Proteintech Group, U.S.A.). An anti-actin antibody (1:1000; Proteintech Group, U.S.A.) was used as loading controls. Immunoreactive proteins were visualized using enhanced chemiluminescence (ECL) and the signals were quantified by densitometry with a Western blotting detection system (Quantity One, Bio-Rad, U.S.A.).

### Statistical analysis

All values were expressed throughout as mean ± SEM. The statistical significance of differences between the various groups was assessed using one-way ANOVA followed by a post hoc test. Data of two groups were analyzed by unpaired t test. A value of P <0.05 was considered statistically significant using SPSS 13.0 Statistical Software.
